# The Model of Crohn’s Disease on Large Laboratory Animals — Pigs

**DOI:** 10.17691/stm2024.16.6.04

**Published:** 2024-12-27

**Authors:** E.N. Fedulova, S.N. Saralov, O.V. Shumilova, N.Yu. Shirokova, K.N. Ilyina, E.A. Farafontova, Yu.P. Rubtsova, M.N. Egorikhina

**Affiliations:** MD, DSc, Head of the F.D. Agafonov Pediatric Department; Head of Pediatric Unit No.1, University Clinic; Privolzhsky Research Medical University, 10/1 Minin and Pozharsky Square, Nizhny Novgorod, 603005, Russia; MD, PhD, Endoscopist, Consulting and Diagnostic Department, University Clinic; Privolzhsky Research Medical University, 10/1 Minin and Pozharsky Square, Nizhny Novgorod, 603005, Russia; Pediatrician, Pediatric Unit No.1, University Clinic; Privolzhsky Research Medical University, 10/1 Minin and Pozharsky Square, Nizhny Novgorod, 603005, Russia; PhD, Senior Researcher, Department of Pathological Anatomy; Privolzhsky Research Medical University, 10/1 Minin and Pozharsky Square, Nizhny Novgorod, 603005, Russia; Tutor, F.D. Agafonov Pediatric Department; Privolzhsky Research Medical University, 10/1 Minin and Pozharsky Square, Nizhny Novgorod, 603005, Russia; Biologist, Laboratory of Biotechnologies, University Clinic; Privolzhsky Research Medical University, 10/1 Minin and Pozharsky Square, Nizhny Novgorod, 603005, Russia; PhD, Researcher, Scientific Laboratory of Cell Technologies; Privolzhsky Research Medical University, 10/1 Minin and Pozharsky Square, Nizhny Novgorod, 603005, Russia; PhD, Head of the Scientific Laboratory of Cell Technologies; Privolzhsky Research Medical University, 10/1 Minin and Pozharsky Square, Nizhny Novgorod, 603005, Russia

**Keywords:** Crohn’s disease, a model of ulcerative defects on large animals, endoscopic loop

## Abstract

Dissatisfactory results of treating inflammatory bowel disease require the development of new modern methods of therapy.

**The aim of the study** is to create a model of Crohn’s disease on large animals, in which ulcerative defects can be formed, for testing new treatment techniques and assessing their effectiveness by endoscopic and morphological methods.

**Materials and Methods:**

The model was created and tested on 12 castrated male pigs (hybrids between the Wiesenau and the Vietnamese black potbellied pigs), aged 6 months. The animals were manipulated under general sedation in the operating room of the SPF-vivarium for large laboratory animals at Privolzhsky Research Medical University (Russia). Endoscopic techniques and a highfrequency electrosurgical apparatus were used to create the required defects. The results were assessed endoscopically and with histological and morphometrical techniques on days 7, 14, and 21.

**Results:**

The morphological examination of the pigs’ intestinal mucous membrane has detected the signs typical of Crohn’s disease, demonstrating the possibility of using pigs as a model of ulcerative defects in Crohn’s disease.

**Conclusion:**

This model of Crohn’s disease on large animals (pigs, in particular) significantly widens the borders of using new treatment techniques at the preclinical stage and will improve therapy effectiveness in patients with this disease reducing the risk of surgical intervention.

## Introduction

Crohn’s disease and ulcerative colitis are the two main phenotypes of inflammatory bowel disease (IBD) and chronic idiopathic inflammatory disease of the gastrointestinal tract [1–3]. Patients suffer from a continuously recurrent course of the disease and are often administered immunomodulators and/or monoclonal antibodies to eliminate the symptoms [4–7]. However, no primary response, loss of secondary response, and an increased risk of serious opportunistic infections are frequently observed. Moreover, despite the development of new genetically engineered therapies, up to 30% of patients with ulcerative colitis and 80% of patients with Crohn’s disease still require surgical intervention to alleviate symptoms associated with progressive damage to the intestinal wall [8–9]. Crohn’s disease is characterized by a chronic and recurrent inflammation of the intestinal tract. Its pathogenesis is not completely clear. It is assumed that there is impaired regulation of the innate and adaptive immune systems, with genetic impacts and environmental factors interfering to cause the development of the disease [10]. Despite the appearance of new genetic engineering techniques in therapy, 30% of patients with ulcerative colitis and 80% with Crohn’s disease still need surgical intervention to alleviate the symptoms caused by progressing damage of the intestinal wall [8, 9].

Crohn’s disease is characterized by chronic and/ or recurrent inflammation of the intestinal tract. Its pathogenesis is not fully understood. Regulation disorder of the innate and adaptive immune system, genetic influence, and environmental factors are supposed to interfere with the development of the disease [10]. Despite the existing treatment methods, still there is a considerable part of patients who fail to achieve remission. The development of new methods is required to overcome the difficulties in IBD treatment [11, 12].

Thus, in connection with the increased incidence of IBD, aggravation in the course of the disease, and the growing number of patients requiring surgical intervention, there is an urgent need to study alternative techniques of treating this disease. To solve the task, it is important to create models of the disease on large animals, in which typical ulcerative defects can be created, enabling easy testing of new treatment techniques and the assessment of their effectiveness by endoscopic and morphological approaches.

Current literature describes mainly the chemical techniques to create models of IBD, since their endoscopic image reflects more exactly the signs of ulcerative colitis. This approach made it possible to develop several types of animal models of colitis induced pharmacologically by dextran sulphate sodium (DSS), trinitrobenzene sulfonic acid (TNBS) or acetic acid (AA) [13, 14]. DSS and TNBS are often used in combination with ethanol in such models [15]. Chemically induced models of IBD are relatively simple and can be implemented in a variety of animal types. However, such chemical damage to the intestinal barrier leads to a selflimiting inflammatory response — an acute reaction rather than a chronic disease. Thus, these models are more relevant for the studies of acute inflammation. They may provide only limited information on the IBD pathogenesis and help rather tentatively extrapolate the results to the processes occurring in humans [14].

The use of acetic acid is one of the most widely used techniques to model erosive and ulcerative lesions of the intestinal mucous membrane. As a result of rectal administration of 1 ml of 4% acetic acid into the intestinal cavity to a depth of 8 cm, the following clinical (bloody diarrhea, weight loss) and morphological signs (intense inflammatory response, characterized by massive bleeding, ulceration, thinning of the intestinal wall, a decreased number of crypts, neutrophilic infiltration) are observed [16, 17]. However, as with other techniques of the chemical impact, the resulting model is characterized by a persistent lesion, which is more typical of ulcerative colitis.

Moreover, the above models of ulcerative defects are most commonly used on small laboratory animals, mainly rodents [18, 19]. This prevents an intravital endoscopic examination and dynamic assessment (including morphological assessment) of the effectiveness of the proposed treatment techniques.

**The aim of the study** is to create a model of ulcerative defects of the intestinal wall typical of Crohn’s disease on large animals with the possibility of endoscopic and morphological monitoring of the healing process.

## Materials and Methods

The study was approved by the Ethics Committee of Privolzhsky Research Medical University (Russia) on March 14, 2022 (protocol No.04). Animals housing and the conducted experiments complied with the European Convention for the Protection of Vertebrate Animals used for Experimental and Other Scientific Purposes (Strasburg, 2006).

Manipulations with the animals were performed in the operating-room of the SPF-vivarium for large laboratory animals at Privolzhsky Research Medical University.

The following preparations were used:

Zoletil 100 (Virbac Sante Animale, France);
XylaVet 2% (Pharmamagist Kft., Hungary);
Propofol-Lipuro 5 mg/kg (B. Braun Melsungen AG, Germany);
10% buffered formalin (рН 7.2–7.4) (LLC “ErgoProduction”, Russia).
The equipment used in our study is listed below:
Automated Slide Stainer Gemini AS for staining with hematoxylin and eosin (Thermo Fisher Scientific, USA);
Mindray Veta 5 stationary ultrasound machine (Mindray, China);
Mindray uMEC12 Vet advance veterinary patient monitor (Mindray, China);
Fujinon ЕС-530WL video colonoscope (Fujifilm, Japan);
Endoscopy stand Fujinon System4400 Processor (Fujifilm, Japan);
HF electrosurgical unit BOWA 901-011 (BOWAelectronic, Germany);
Digital video recorder TEAC UR-4MD (TEAC Corporation, Japan);
MTW biopsy forceps (MTW Endoskoрie, Germany);
MTW polypectomy snare (MTW Endoskoрie, Germany);
MTW injection (MWT Endoskoрie, Germany);
Electrosurgical unit Olympus UES-10 (Olympus, Japan);
Morphometric complex Leica DMR (Leica, Germany);
Excelsior ES processor (Thermo Fisher Scientific, USA);
HistoStar tissue embedding system (Thermo Fisher Scientific, USA);
Microm HM 325 rotary microtome (Thermo Fisher Scientific, USA);
Nikon Eclipse E400 microscope (Nicon, Japan) with Nikon DXM1200 digital camera (Nicon, Japan) and ACT- 1 software, version 2.12, and Videotest- Morphology 5.0 (LLC VideoTest, Russia);
Pannoramic histoscanner (3DHISTECH, Hungary).

### Animals

12 castrated male pigs (hybrids between the Wiesenau and Vietnamese black potbellied pigs) aged 6 months were used in the experiment.

### Animal sedation

Formation of the mucosal defect, endoscopic examination, and material sampling for morphological examination were conducted under combined pain relief. The animals were sedated by intramuscular injection of Zoletil 100 (6 mg/kg) and XylaVet 2% (0.3 mg/kg). After sedation, intubation with synchronized intermittent mandatory ventilation (SIMV) of the lungs was performed together with infusion support with Propofol-Lipuro (5 mg/kg), and Zoletil 100 (2 mg/kg).

***Animal health was controlled*** by professional anesthesiologists-reanimatologists. Blood pressure, heart rate, partial oxygen tension in the blood, respiration control and lung ventilation were continuously monitored during the endoscopic intervention using the stationary ultrasound unit and veterinary monitor.

### Histological investigations

The biopsy material obtained during the endoscopic examination was fixed in 10% buffered formalin (pH 7.2–7.4) and subject to a standard histological treatment (dehydration, dewaxing) using the Excelsior ES processor. After the histological treatment, paraffin blocks were made using the HistoStar embedding station. Serial 4–6 μm thick sections were made using the Microm HM 325 microtome and then stained with hematoxylin and eosin. Histological preparations were viewed and photographed on the Nikon Eclipse E400 microscope using the Nikon DXM1200 camera and АСТ-1, v. 2.12, and Videotest- Morphology 5.0 software.

The morphometric technique was used for the objective study of the rectal mucosa structure. Cells were counted in 10 fields of view with a 90× objective and a 10× eyepiece, and the absolute number of cells per 1 mm^2^ was calculated.

### Formation of ulcerative defects of the intestinal wall

The animal was sedated. Before creating the model, the pig’s colon was endoscopically examined in order to assess the state of the intestinal mucous membrane and collect the biopsy material. Preliminary preparation of the pig before anesthesia was not conducted. The animals followed the usual regimen of eating and drinking, no bowel cleaning with enemas was performed. The rectum was manually (with fingers) cleaned to a depth of 8–10 cm. An area of the native mucous membrane was selected under the endoscopic control and captured with the endoscopic loop during peristaltic contraction. The intestinal wall was resected and coagulated up to the muscle layer in a mixed mode of electroexcision using a current of 5.0–6.0 A. The current caused ulcerative defects of the intestinal wall similar in depth and clear boundaries to those typical of Crohn’s disease.

### Statistical analysis

Data obtained were processed using SPSS Statistics v. 23.0 software (StatSoft Inc., USA). The Kolmagorov–Smirnov test was applied to assess the compliance of the sample with the law of probability distribution, which was amounted to less than 0.05 of the coefficients of asymmetry and excess. The median, low and upper quartiles (Mе [Q1; Q3]) were used for data description. Initially, the results of the study were processed using the non-parametric Friedman test to evaluate statistical significance between the groups of comparison, then the non-parametric Mann–Whitney test was employed for paired comparison between the group with adaptation norm (healthy animals) and four groups with various observation periods. Taking into consideration the Bonferroni corrections, the standard critical significance level of p<0.0125 was adopted.

## Results

The results of the ulcerative defect formation were comparable in all 12 animals. As an example, a detailed description of the results of the defect formation in two pigs is presented below.

Examples of the applied techniques for the creation of a Crohn’s disease model on large laboratory animals such as pigs

### Example 1

A 6-month old castrated male pig (a crossbreed of Wiesenau with a Vietnamese black potbellied pig) weighing 28,900 g.

**Figure 1.**
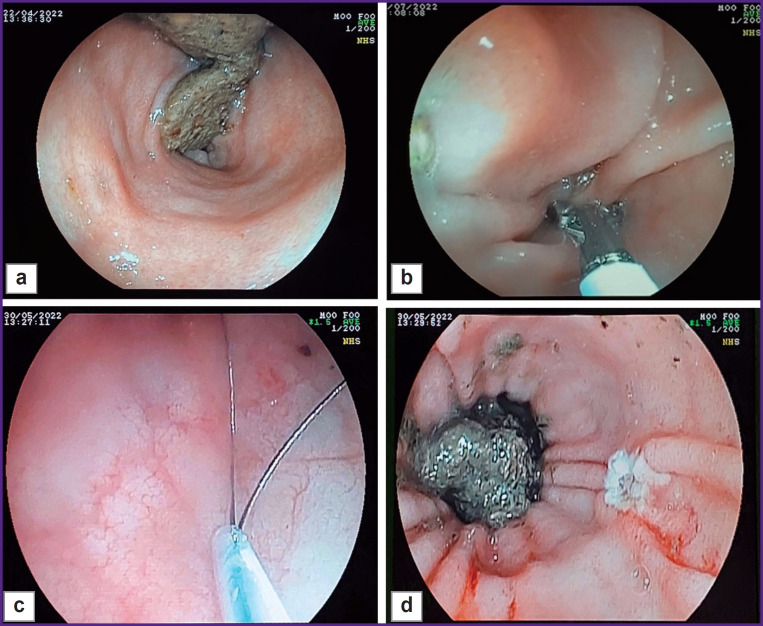
Formation of the ulcerative defect in the rectum (example 1), endoscopic examination: (а) unchanged rectal mucous membrane; (b) forceps biopsy of the rectal mucous membrane; (c) loop capture of the rectal mucous membrane; (d) created ulcerative defect on the rectal mucous membrane having almost a rounded shape with hyperemic edges and a dense whitish deposit (fibrin); 0.7×0.8 cm in size

The animal was sedated without prior preparation as specified above, the rectum was manually cleaned to a depth of 10 cm (removal of feces). The endoscopic examination showed the satisfactory rectal cleaning (Figure 1 (a)). A biopsy sample was taken from the intact colonic mucous membrane (Figure 1 (b)) capturing 10 mm of mucous membrane with a loop on the peristalsis. The loop was then tightened, a section of the mucous membrane was removed, and the damaged area exposed to a 5 A current (Figure 1 (c)). The rectal mucous membrane was examined to detect the presence of the ulcerative defect (Figure 1 (d)) and the removal of the dissected area. The size of the ulcerative defect was 10 mm. The day after the ulcerative defect modeling (day 1), the biopsy material was collected for morphological evaluation of the model obtained.

The biopsy sample of the mucous membrane 1 day after the defect creation looked in the following way:

the surface epithelium was absent;
eroded defects could be determined;
crypts with an irregular arrangement were visible, their number reduced; the number of goblet cells in some of the crypts was sharply decreased up to their complete elimination;
the lamina propria of the mucous membrane demonstrated marked diffused lymphoplasmacytic infiltration with a significant number of neutrophilic leukocytes forming cluster foci including their dissemination into the epithelium of many crypts with the formation of crypt abscesses, followed by the destruction of some crypts;
the walls of the microvessels were loosened, contained swollen endothelial cells, showing partial infiltration by neutrophils; the latter, inter alia, could also be determined in the lumen of some vessels;
in some areas, the muscular plate was fragmented with sporadic foci of neutrophilic infiltration;
pronounced inflammatory infiltration with a significant number of neutrophils was seen in the submucosa;
vessels with swollen endotheliocytes were observed; the lumens of some of them were expanded and contained neutrophils.

**Figure 2.**
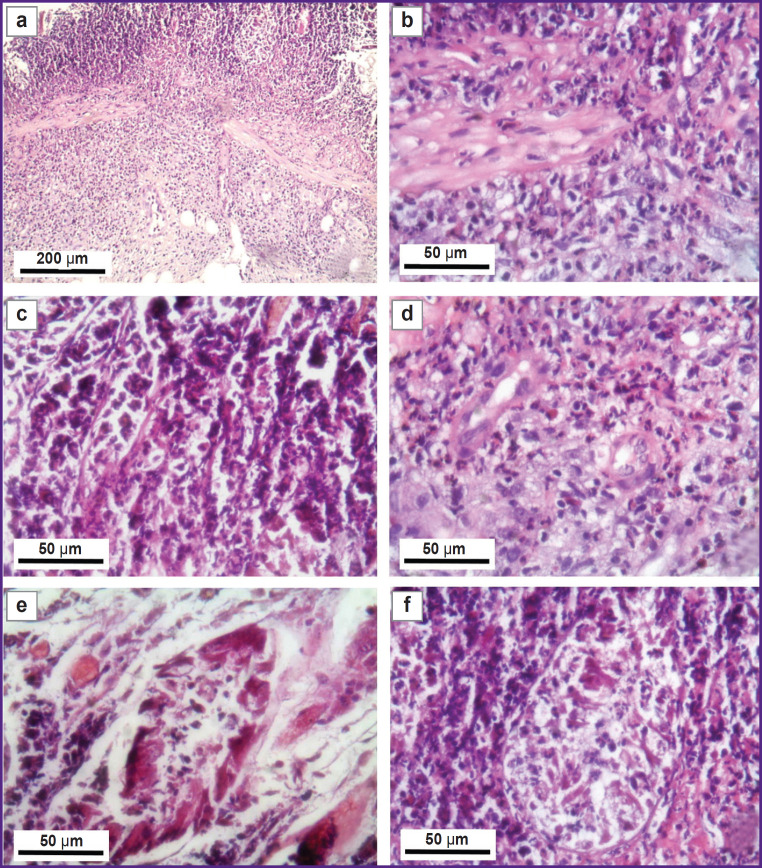
A morphological pattern of the changes at the site of the rectal defect on day 1, staining with hematoxylin and eosin (example 1): (а) pronounced neutrophilic infiltration of all layers of the pig’s rectum (submucosa, muscular lamina, mucous membrane), 40×; (b) fragmentation and marked infiltration of the muscular mucosal lamina by neutrophilic leukocytes, 400×; (c) accumulations of neutrophilic leukocytes in the lumens of microvessels in the intercryptal space of the mucous membrane, 400×; (d) swollen endotheliocyte nuclei; greatly thickened walls of microvessels, partly infiltrated by polymorphonuclear leukocytes concurrently with significant neutrophilic infiltration of the submucosal layer, 400×; (e) tissue edema and lysis near a dying crypt, 100×; (f) destroyed crypt with a large crypt abscess in the bulk of neutrophilic granulocytes in the intercryptal space of the mucous membrane, 400×

The morphological picture of the obtained changes at the site of the defects was as follows: neutrophilic infiltration of all layers of the intestinal wall (Figure 2 (a)), fragmentation and expressed infiltration of neutrophilic leukocytes into the muscular lamina of the mucous membrane (Figure 2 (b)), vessels of the microcirculation bed with a significant content of neutrophils in the vessel lumens (Figure 2 (c)), swollen endothelium, thickened vascular walls of the microvessels surrounded by pronounced inflammatory infiltration (Figure 2 (d)), tissue edema and lysis near the dying crypt (Figure 2 (e)), a destroyed crypt with a large crypt abscess in the bulk of neutrophilic granulocytes in the intercryptal space of the mucous membrane (Figure 2 (f)).

All parameters of the morphological pattern observed corresponded to those of the ulcerative defects in Crohn’s disease [20–21].

**Figure 3.**
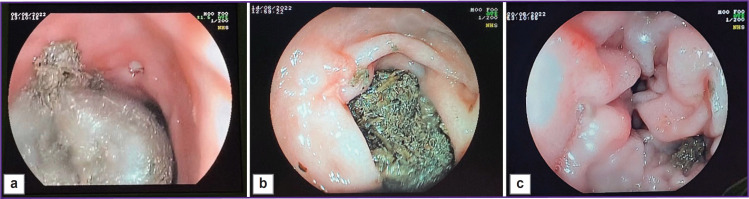
The created defect at various stages of the study (example 1): (а) day 7, the ulcerative defect 0.5 cm in size, of irregular shape, with a hyperemic base and a fragmented grayish deposit (fibrin); (b) day 14, a scarring ulcer, longitudinal in shape, about 0.5 cm in size, with focal marginal hyperemia and a whitish deposit in the center (fibrin) with slight convergence of the mucous membrane; (c) day 21, formation of a red scar 0.5 cm in size

The defect was controlled on days 7, 14, and 21 (Figure 3). The figure shows that by day 21 of the study, a tender scar had formed at the site of the ulcerative defect.

The animal’s health during the experiment was satisfactory, no deterioration in appetite or bowel habits was observed.

### Example 2

A 6-month old castrated male pig (a crossbreed of the Wiesenau and Vietnamese black potbellied pig) weighing 28,850 g.

**Figure 4.**
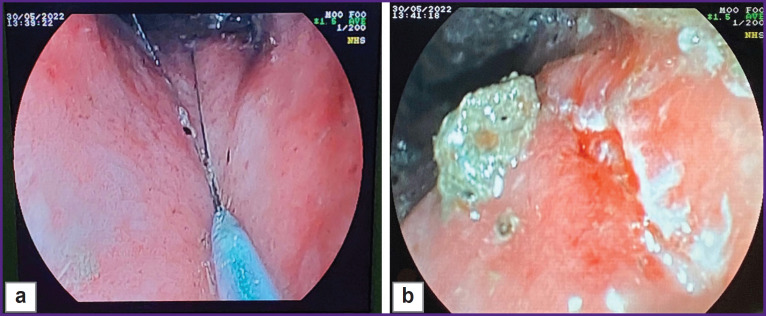
Formation of the rectal ulcerative defect (example 2), endoscopic examination: (а) capture of the mucous membrane by the endoscopic loop; (b) formation of the 0.11 cm ulcerative defect

The animal was given general anesthesia using Zoletil 100 agent without prior preparation, the rectum was manually cleaned to a depth of 8 cm (manual removal of feces) and the endoscope was introduced. When the endoscopic examination showed a satisfactory rectal cleaning, the mucous membrane was captured with an endoscopic loop (Figure 4 (a)). The intestinal wall was dissected and coagulated to the depth of the muscle layer in a mixed mode of electrical excision using a 6.0 A current. The defect obtained by the above manipulations was 11 mm in size (Figure 4 (b)).

The day after modeling the ulcerative defect, a biopsy sample was taken for morphological assessment. The bioptate of the mucous membrane after the formation of the defect looked as follows:

a large area of the surface epithelium had erosive changes; no goblet cells were noted;
the crypt layer was sharply narrowed to the point of complete crypt disappearance;
in the lamina propria of the mucous membrane, diffuse pronounced lymphoplasmacytic infiltration with a significant number of polymorphonuclear leukocytes was noted;
the microvessels had a swollen endothelium, the lumens of many vessels contained neutrophilic leukocytes;
diffuse pronounced inflammatory infiltration was also found in the submucous layer with a significant number of polymorphonuclear leukocytes, in some visual fields they penetrated into the muscular lamina.

**Figure 5.**
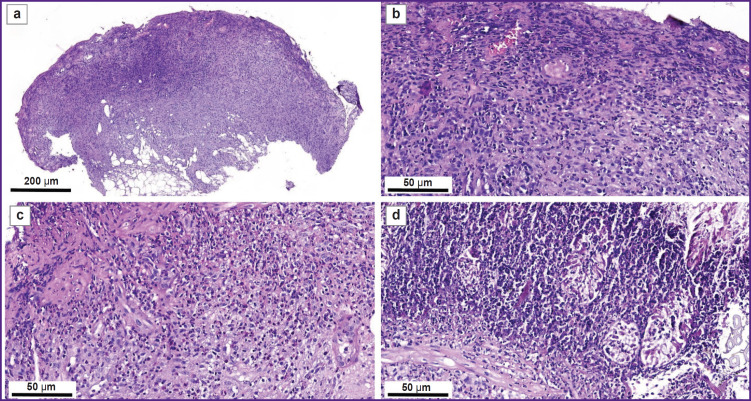
A morphological pattern of the changes at the site of the rectal defect, day 1, staining with hematoxylin and eosin (example 2): (а) eroded surface of the mucous membrane with a pronounced neutrophilic infiltration, a general view of the biopsy sample, 40×; (b) significant neutrophilic leukocyte infiltration of the rectal mucous membrane with complete destruction of the crypt layer, 400×; (c) neutrophilic granulocyte expansion of the muscular lamina, reaction of the microcirculatory bed, vasculitis (greatly thickened walls, swollen nuclei of endotheliocytes, PMNs in the vessel lumens), 100×; (d) multiple crypt abscesses with destruction of the crypts together with a significant infiltration by neutrophilic leukocytes, destruction of the muscular lamina and submucosal layer by neutrophilic leukocytes, 400×

Figure 5 shows the eroded surface of the mucous membrane with a pronounced neutrophilic infiltration of all layers of the intestinal wall (Figure 5 (a)); the cells of the inflammatory infiltrate destroying the crypts (Figure 5 (b)); inflammatory infiltration of the muscular lamina with the penetration into the submucous layer, vasculitis (Figure 5 (c)); multiple crypt abscesses together with dying crypts, the destruction and lysis of the muscular plate and the submucous layer concurrently with significant infiltration by neutrophilic leukocytes (Figure 5 (d)).

The morphological study of the mucous membrane has revealed the signs typical of Crohn’s disease [20]: deeply eroded and ulcerative defects involving all layers of the intestinal wall; pronounced inflammatory infiltration in the submucosa and a significant number of polymorphonuclear leukocytes penetrated into the muscular lamina and absence of goblet cells.

Concurrently, a noticeable decrease of the cell number in the fibroblastic differon was seen. Besides, there was almost a 2-fold decrease in the number of cells of the mast cell population with the predominance of young cell forms having a low synthetic activity. Furthermore, a higher content of macrophages relative to the normal condition was noted, which, in this case, ensure the elimination of the degenerated components of the mucosal lamina propria (MLP) and extracellular matrix.

The analysis of the microcirculatory bed in the MLP of the pig’s rectum on day 1 of the follow-up indicated a sharp circulatory disorder. There was a reduction in the MLP vessels both in the superficial and deeper layers.

**Figure 6.**
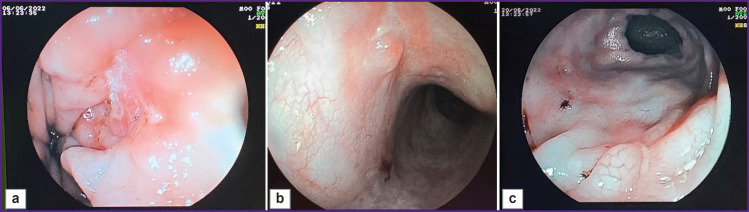
The created defect (example 2) at various stages of the study: (а) day 7, the ulcerative defect of about 1 cm in diameter with hyperemic edges and a whitish fragmented deposit (fibrin); (b) day 14, a scarring ulcer, longitudinal in shape, about 0.8–1.0 cm in size, with focal marginal hyperemia and a whitish deposit in the center (fibrin) with a slight convergence of the mucous membrane; (c) day 21, scar of longitudinal shape, approximately 0.5–0.8 cm in size, with marginal hyperemia and a slight convergence of the mucous membrane, transition from the red scar stage to the stage of a white scar

Further endoscopic and morphological investigation was performed on days 7, 14, and 21 of the experiment (Figure 6). Three weeks after the defect modeling, a small scar remained at the site of the original defect (Figure 6 (c)).

The state of the animal throughout the experiment was satisfactory, there was no deterioration in the appetite or bowel habits.

### The analysis of the histological material in dynamics

The analysis of the histological material on day 7 showed a pattern of a marked inflammatory reaction in all experimental animals (see the Table). For instance, there was a more than 1.5-fold increase of the cell infiltrate density, which reached its maximum due to a sharp growth in the number of the lymphoplasmacytic cells with the presence of a significant number of polymorphonuclear leukocytes. The number of lymphoplasmacytic series cells had increased by more than 2.6 times, and the number of polymorphonuclear leukocytes was almost 3 times higher compared to the unaffected areas of the mucous membrane. The occurrence of active neoangiogenesis with the appearance of multiple thin-walled capillary-type vessels was seen.

**Table:** Morphometric indicators of the pig’s rectal mucosa in norm and in different observation periods after defect creation, Me [Q1; Q3]

Indicators	Norm	Day 1	Day 7	Day 14	Day 21	p
	1	2	3	4	5	
Number of cells per 1 mm^2^	8828	11 147	12 824	10 807	9694	p_1–2_=0.0123; p_1–3_≤0.001
	[8594; 9062]	[10,808; 11,486]	[12,437; 13,211]	[10,170; 11,444]	[9352; 10,036]	p_1–4_≤0.001; p_1–5_=0.226
Lymphocytes	2306	4430	4086	3053	2810	p_1–2_≤0.001; p_1–3_=0.011
	[2160; 2452]	[4252; 4608]	[3919; 4253]	[2840; 3266]	[2437; 3211]	p_1-4_=0.0125; p_1–5_=0.12
Plasmocytes	989	2201	2997	1581	943	p_1–2_=0.0116; p_1–3_≤0.001
	[936; 1042]	[2050; 2352]	[2794; 3200]	[1444; 1718]	[901; 985]	p_1–4_=0.0120; p_1–5_=0.051
Macrophages	407	592	672	577	488	p_1–2_=0.0115; p_1–3_≤0.001
	[369; 445]	[549; 635]	[618; 726]	[533; 621]	[457; 519]	p_1–4_=0.0120; p_1–5_=0.045
Fibroblasts	2574	1543	1980	2791	2774	p_1-2_≤0.001; p_1-3_=0.051
	[2428; 2720]	[1406; 1680]	[1856; 2104]	[2640; 2942]	[2637; 2911]	p_1–4_=0.047; p_1–5_=0.095
Fibrocytes	1204	902	991	1298	1287	p_1-2_=0.074; p_1-3_=0.15
	[1093; 1315]	[838; 966]	[916; 1066]	[1192; 1404]	[1173; 1401]	p_1–4_=0.21; p_1–5_=0.19
Eosinophilic granulocytes	266	408	479	294	309	p_1-2_=0.0081; p_1-3_≤0.001
	[234; 298]	[360; 456]	[445; 513]	[262; 326]	[285; 333]	p_1–4_=0.091; p_1–5_=0.075
Neutrophilic granulocytes	202	644	618	327	271	p_1–2_≤0.001; p_1–3_=0.0122
	[184; 220]	[585; 703]	[567; 669]	[303; 351]	[250; 292]	p_1–4_=0.086; p_1–5_=0.098
Labrocytes	486	241	349	381	405	p_1-2_≤0.001; p_1-3_=0.073
	[455; 517]	[218; 264]	[322; 376]	[352; 410]	[367; 443]	p_1–4_=0.061; p_1–5_=0.26
Vessels	394	186	652	505	406	p_1–2_=0.0125; p_1–3_=≤0.001
	[370; 418]	[158; 214]	[603; 701]	[464; 546]	[369; 443]	p_1–4_=0.0114; p_1–5_=0.092

In the period from the end of day 14 to day 21, a gradual decrease in the cell infiltrate density was observed in the micropreparations of the rectal MLP. This was due to a decrease in the number of plasma cells, lymphocytes, and in the number of cells with aggressive potential: a 1.8-fold decrease in polymorphonuclear leukocytes and a consistent increase in fibroblast cells and in the mast cell population with the growth in active mature cell forms. In this situation, the number of vessels of microcirculation tended to decrease and reached the indicators close to those of the normal condition (see the Table).

### Advantages of the model

**Figure 7.**
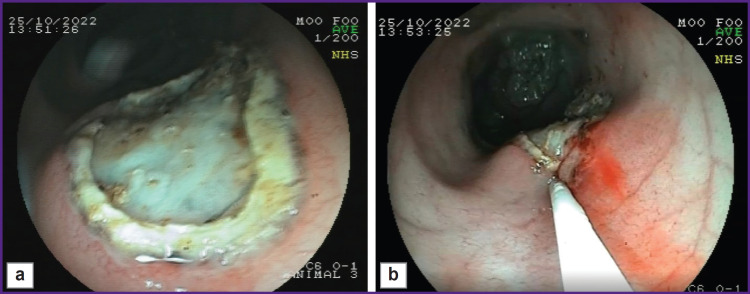
Endoscopic and morphological dynamic monitoring of the pig’s rectal defect with the assessment of the manipulation effectiveness: (а) ulcerative defects of the intestinal wall formed by coagulation; (b) introduction of the medication through the endoscopic injector into the edge of the ulcerative defect

The described model enables the creation of intestinal ulcerative defects corresponding to the morphological pattern of Crohn’s disease. It should be noted that forming a defect with clear boundaries using an endoscopic loop facilitates the investigation and visualization of the defect during subsequent studies (Figure 7 (a)). A defect with a diameter of at least 10 mm is sufficient for testing medications and medical substances for a 3-week period after its creation (see Figure 3 (c) and Figure 6 (c)). Moreover, it is possible to conduct endoscopic and morphological dynamic monitoring of the defect and assess manipulation effectiveness (for example, Figure 7 (b) shows the introduction of medication through the endoscopic injector into the edge of the ulcerative defect). Furthermore, the defect remains for 3 weeks making it possible to assess the ulcerative defect during this period (see Figure 3 (c) and Figure 6 (c)).

## Conclusion

The proposed technique enables the creation of ulcerative defects of the intestinal wall, corresponding in depth (to the muscle wall) and clear boundaries to the standard pattern of ulcerative defects typical of Crohn’s disease: isolated ulcerative defects with clear edges, sometimes undermined, on the intact mucous membrane. The typical morphological signs of these ulcers include deep erosive and ulcerative defects extending to all layers of the intestinal wall, pronounced inflammatory infiltration in the submucosa with a significant number of polymorphonuclear leukocytes that penetrate into the muscular lamina, and the absence of goblet cells.

The clear boundaries of the defects in the presented model facilitate the introduction of medications and monitoring of their effectiveness during regeneration. This large-animal model significantly expands the perspectives for the implementation of new treatment techniques at the preclinical stage of their development and helps improve treatment effectiveness in patients with Crohn’s disease, thus decreasing the need for surgical intervention.
